# Clinical and radiographic factors associated with surgical approach selection in total hip arthroplasty: a preliminary machine learning analysis

**DOI:** 10.3389/fsurg.2026.1891101

**Published:** 2026-07-21

**Authors:** Meng Li, Yuanye Ge, Dalin Wang, Peng Li, Haoyan Sun, Xiang Zhang, Zhe Wang

**Affiliations:** 1 College of Clinical Medicine, Beihua University, Jilin City, Jilin, China; 2 Department of Orthopedics, Tengzhou Orthopedic Hospital, Tengzhou, Shandong, China; 3 Orthopedic Center, Beihua University Affiliated Hospital, Jilin City, Jilin, China

**Keywords:** direct anterior approach, machine learning, posterolateral approach, radiographic assessment, total hip arthroplasty, XGBoost

## Abstract

**Objective:**

To investigate the clinical and radiographic factors associated with surgeon selection of the direct anterior approach (DAA) versus the posterolateral approach (PLA) in total hip arthroplasty (THA), and to explore whether machine learning methods can characterize historical surgical selection patterns.

**Methods:**

A retrospective analysis was performed on 98 patients who underwent primary THA at two institutions, including 61 patients in the PLA group and 37 patients in the DAA group. Patient demographics and preoperative radiographic parameters were collected. Baseline comparisons and univariate logistic regression analyses were conducted using SPSS. A machine learning classification model reflecting historical surgeon-selected approaches was constructed using the XGBoost algorithm, and its performance was evaluated via receiver operating characteristic (ROC) curves, five-fold cross-validation, SHAP analysis, and calibration curves.

**Results:**

Patients in the DAA group exhibited significantly higher age, soft tissue thickness, neck-shaft angle (NSA), and femoral offset compared to those in the PLA group (all *P* < 0.05). Univariate logistic regression revealed that age, NSA, and femoral offset were significantly associated with DAA selection. The XGBoost model achieved an area under the curve (AUC) of 0.938 and an accuracy of 90.0% on the test set, with a mean AUC of 0.897 via five-fold cross-validation. SHAP analysis identified osteoporosis, Dorr classification, age, and NSA as key contributors to model predictions. The model exhibited good calibration, as indicated by a Brier score of 0.0898.

**Conclusion:**

The present study identified several clinical and radiographic factors associated with historical surgeon selection of DAA versus PLA in THA. The machine learning model demonstrated the ability to characterize real-world surgical selection patterns, although it should not be interpreted as recommending the optimal surgical approach for individual patients.

## 1 Introduction

Total hip arthroplasty (THA) is one of the most successful surgical procedures for the treatment of end-stage hip disease. It is currently widely utilized in the management of femoral head necrosis, osteoarthritis, femoral neck fractures, and developmental dysplasia of the hip, and is recognized as one of the most cost-effective interventions in joint surgery (1). A major focus of contemporary THA research is to further reduce soft tissue injury while ensuring implant stability. The most commonly employed surgical approaches in clinical practice include the posterolateral approach (PLA) and the direct anterior approach (DAA). The PLA, owing to its specific advantages, has become one of the most widely utilized classic approaches, particularly suitable for complex deformities and revision cases (2). However, this approach necessitates incision of the posterior capsule and partial division of the external rotator muscles, which may increase the risk of postoperative dislocation (3). In contrast, the DAA utilizes the intermuscular plane to access the hip joint without detaching major muscle groups, thereby reducing soft tissue damage and facilitating early functional recovery (4). Studies have demonstrated that patients undergoing DAA may experience advantages in terms of early postoperative pain, gait recovery, and length of hospital stay (5).

Currently, the results of studies comparing the advantages and disadvantages of DAA and PLA remain controversial, and a unified, definitive criterion for THA approach selection has yet to be established. Nevertheless, the selection of a THA approach is not entirely random; rather, it is influenced by a combination of multiple patient-specific characteristics (6). Furthermore, radiographic parameters such as femoral neck anteversion, acetabular coverage, and proximal femoral morphology may also affect the difficulty of intraoperative exposure and the accuracy of implant placement. Research on the relationship between these preoperative factors and surgical approach selection remains relatively limited, particularly regarding quantitative predictive models based on real-world clinical data. Compared with traditional statistical methods, machine learning is capable of simultaneously processing complex nonlinear relationships among multidimensional variables, thereby enabling analysis of complex relationships among multidimensional clinical variables and real-world surgical decision patterns. Previous studies have attempted to apply artificial intelligence-assisted systems to preoperative planning for THA and have achieved favorable outcomes in terms of implant positioning (7). However, machine learning studies specifically focused on predicting surgical approach selection in THA are lacking, particularly those integrating both clinical characteristics and radiographic parameters into models reflecting real-world surgeon selection patterns. Based on this background, the present study aims to employ a multicenter retrospective case series to collect general clinical data and preoperative radiographic parameters. A machine learning-based prediction model for THA surgical approach selection will be constructed to identify key factors influencing the choice between DAA and PLA, and to analyze the factors associated with historical surgical approach selection in routine clinical practice.

## 2 Methods

### 2.1 Study design and patient source

**Figure 1:**
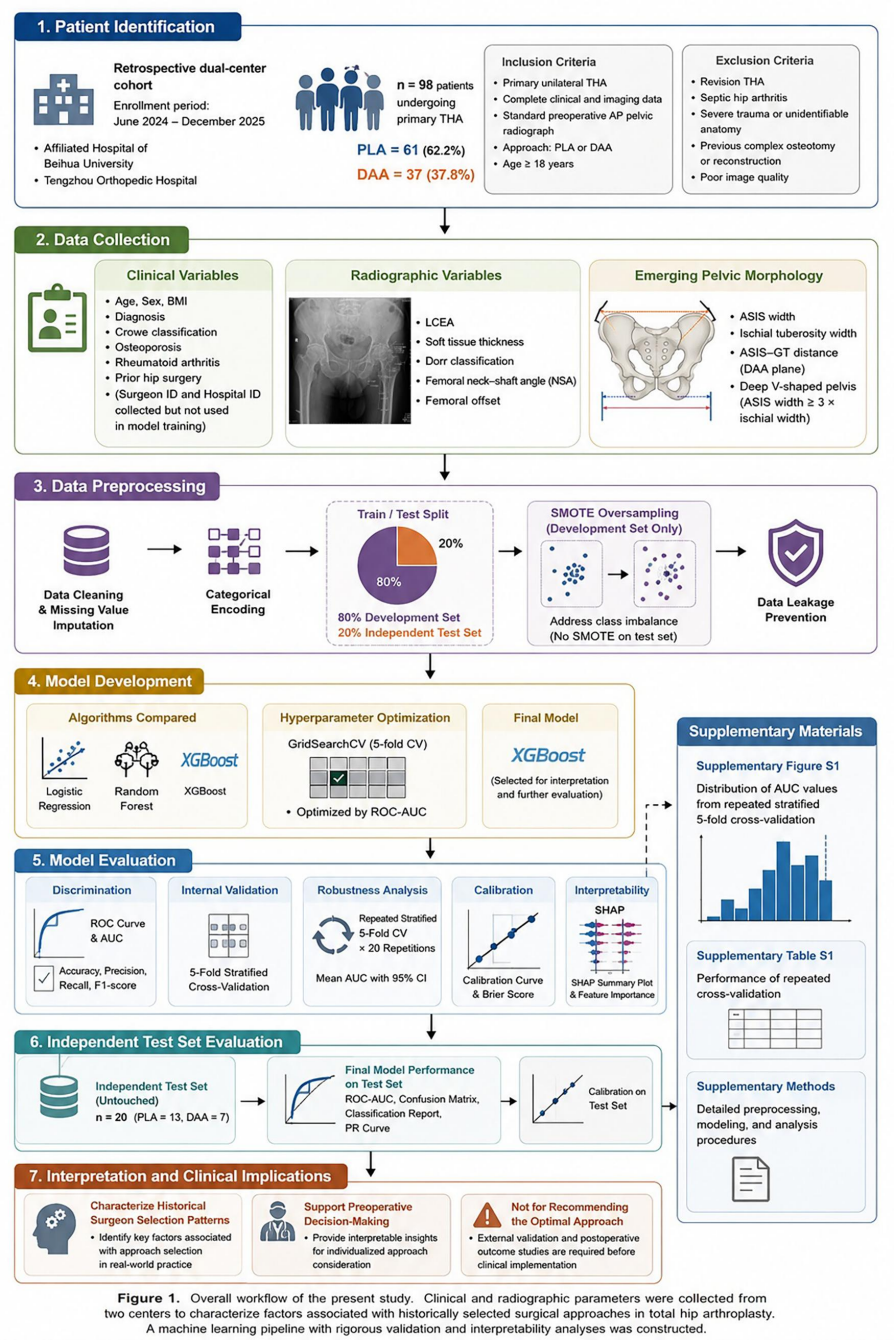
Overall workflow of the present study. Clinical and radiographic parameters collected from two centers were used to analyze factors associated with historically selected surgical approaches in total hip arthroplasty through machine learning-based classification and interpretability analyses.

This study was designed as a retrospective, multi-center retrospective observational analysis incorporating machine learning classification methods. Patient data were collected from the joint surgery centers of two tertiary hospitals. All surgical procedures were performed by two senior orthopedic professors (Level II) with extensive experience in total hip arthroplasty. The enrollment period spanned from June 2024 to December 2025. The study protocol was approved by the Institutional Review Board of the Affiliated Hospital of Beihua University (Approval No.202600080) and Tengzhou Orthopedic Hospital (Approval No.2026001). Given the retrospective design and use of anonymized data, the requirement for written informed consent was waived. All procedures were conducted in accordance with the Declaration of Helsinki. Figure 1 illustrates the complete workflow of this study, encompassing case screening, radiographic measurements, statistical analysis, machine learning model construction, and interpretability analysis.

### 2.2 Inclusion and exclusion criteria

#### 2.2.1 Inclusion criteria

Patients were subjected to primary unilateral THA; complete preoperative clinical and imaging data were available; standard anteroposterior pelvic radiographs were obtained; the surgical approach was explicitly designated as the DAA or PLA; age ≥ 18 years.

#### 2.2.2 Exclusion criteria

Revision THA; septic hip arthritis; unidentifiable pelvic or proximal femoral anatomy due to severe trauma; cases with a history of previous complex osteotomy or reconstructive surgery; poor image quality precluding accurate measurement.

### 2.3 Clinical data collection

General patient information and details of underlying conditions were collected, including: age, sex, body mass index (BMI), disease type, Crowe classification, osteoporosis, rheumatoid arthritis, history of prior hip surgery, surgeon identification number, and hospital center identification number.

### 2.4 Radiographic measurements

All radiographic measurements were performed based on preoperative standard anteroposterior pelvic radiographs. To minimize observer variability, two surgeons with over five years of experience in joint surgery independently performed the measurements, and the mean values were used for subsequent analysis. The measured parameters included: lateral center-edge angle (LCEA), soft tissue thickness, Dorr femoral morphology classification, femoral neck-shaft angle, and femoral offset.

### 2.5 Data preprocessing

All data were imported into the Python environment for analysis. Categorical variables were numerically encoded to meet the input requirements of machine learning models. To mitigate the influence of operator bias and institutional bias on the model, surgeon identifiers and hospital center identifiers were not included in the final model training. Given the class imbalance between DAA and PLA cases, and to prevent data leakage, the dataset was first split into a development set and a test set. The Synthetic Minority Oversampling Technique (SMOTE) was subsequently applied to oversample the minority class in the development set, thereby improving the model's ability to identify the minority class. In addition to machine learning analyses, conventional statistical analyses were performed using SPSS version 27.0. For handling missing data, a completeness check was first conducted for all variables. Continuous variables with a missing rate below 5% were imputed using median imputation, while categorical variables were imputed using mode imputation. Due to the overall low proportion of missing data, multiple imputation was not employed. To avoid potential small-sample bias resulting from the deletion of cases with missing values, all eligible cases were retained for model construction. For categorical variables, label encoding was employed for numerical transformation. Specifically, binary variables such as sex, osteoporosis, rheumatoid arthritis, and history of previous surgery were encoded as 0 and 1; multi-categorical variables such as Dorr classification and Crowe classification were encoded as integers according to the clinical grading order, thereby preserving potential ordinal information. Since XGBoost and Random Forest are tree-based ensemble learning algorithms that are insensitive to variations in variable scale, continuous variables were not standardized, in order to retain the practical physical significance of the original clinical data. In the present study, the outcome label did not represent the objectively optimal surgical approach for individual patients. Instead, the label reflected the actual surgical approach selected by experienced arthroplasty surgeons during routine clinical practice. Therefore, the machine learning model was designed to characterize historical surgeon selection patterns rather than to recommend the best surgical approach. To prevent information leakage, the dataset was first divided into development and independent test sets. SMOTE was applied only to the development set after data partitioning and was not used on the independent test set. The test set remained untouched throughout model development and evaluation.

### 2.6 Establishment of machine learning models

Machine learning modeling in this study was performed in the Python environment. All cases were randomly divided into a development set and a test set at a ratio of 8:2, with stratified sampling employed to ensure consistent label distribution. To enhance the reproducibility of this study, a fixed random seed (random_state = 42) was set to ensure stability and consistency in data partitioning, SMOTE sampling, and model training processes. A classification model was established using the extreme gradient boosting (XGBoost) algorithm. XGBoost is an ensemble learning algorithm based on gradient-boosted decision trees, exhibiting favorable stability and generalization capability in the analysis of structured medical data with small to moderate sample sizes.All machine learning analyses were performed using Python 3.11. The primary libraries included scikit-learn (v1.5.1), xgboost (v2.1.1), imbalanced-learn (v0.12.3), pandas (v2.2.2), NumPy (v2.0.1), and SHAP (v0.46.0).

### 2.7 Model performance evaluation and visualization analysis

The discriminative ability of the model was evaluated using the receiver operating characteristic (ROC) curve and the area under the ROC curve (AUC). Additionally, several metrics, including Accuracy, Recall, and F1-score, were calculated to comprehensively assess model performance. To improve model interpretability, SHAP (SHapley Additive exPlanations) analysis was employed to evaluate the contribution and direction of influence of each variable on the model's predictions. The stability of the model was assessed using 5-fold cross-validation. The discriminative ability of the model was further evaluated using the ROC curve and AUC. Model calibration was assessed using the calibration curve and the Brier score.

### 2.8 Statistical analysis

All statistical analyses were performed using SPSS version 27.0 (IBM Corp., Armonk, NY, USA), while machine learning analyses were conducted in Python 3.11. Continuous variables were first assessed for normality using the Shapiro–Wilk test. Normally distributed variables were expressed as mean ± standard deviation (SD) and compared between groups using the independent-samples t-test. Non-normally distributed variables were presented as median (interquartile range) and analyzed using the Mann–Whitney U test. Categorical variables were presented as frequencies and percentages. Group comparisons were performed using the chi-square test or Fisher's exact test when the expected cell count was less than 5.To identify factors associated with historical selection of the DAA versus the PLA, univariate logistic regression analyses were performed. Variables were entered individually into the regression model, and regression coefficients (β), odds ratios (ORs), 95% confidence intervals (CIs), and corresponding *P*-values were reported. All statistical tests were two-sided, and a *P*-value < 0.05 was considered statistically significant.

## 3 Results

### 3.1 Patient enrollment and baseline characteristics

A total of 98 patients who underwent primary THA were enrolled in this study, including 61 patients subjected to the PLA and 37 patients subjected to the DAA. Baseline characteristics are presented in Table 1. The results showed that patients in the DAA group were significantly older than those in the PLA group [68.62 ± 8.51 years vs. 58.03 ± 10.19 years, (*P* < 0.001)]. Soft tissue thickness was significantly greater in the DAA group compared with the PLA group [23.37 ± 5.37 mm vs. 18.98 ± 5.86 mm, (*P* < 0.05)]. Furthermore, the femoral neck-shaft angle [136.92° ± 7.61° vs. 128.61° ± 8.24°, (*P* < 0.001)] and femoral offset [41.88 ± 4.24 mm vs. 38.14 ± 7.95 mm, (*P* < 0.05)] were significantly higher in the DAA group than in the PLA group. No statistically significant differences were observed between the two groups in terms of BMI, LCEA, or history of prior surgery (*P* > 0.05). These findings suggest that patients receiving different surgical approaches exhibit distinct differences in age, femoral geometry, and soft tissue conditions, indicating that surgeons have already incorporated patient anatomical characteristics into their selection of the surgical approach in clinical practice.

**Table 1:** Patient baseline table incorporated into machine learning.

Variable	PLA group (*n* = 61)	DAA group (*n* = 37)	*P*
Age (years)	58.03 ± 10.19	68.62 ± 8.51	<0.001
BMI (kg/m²)	23.24 ± 3.66	22.99 ± 2.74	0.722
LCEA (°)	35.65 ± 2.91	35.80 ± 2.79	0.801
Thickness (mm)	18.98 ± 5.86	23.37 ± 5.37	<0.001
NSA (°)	128.61 ± 8.24	136.92 ± 7.61	<0.001
Femoral offset (mm)	38.14 ± 7.95	41.88 ± 4.24	0.010

### 3.2 Univariate logistic regression analysis

Univariate logistic regression analysis was conducted to explore factors associated with historical surgeon selection of DAA versus PLA (Table 2). Older age (OR = 1.133, 95% CI: 1.070–1.199, *P* < 0.001), higher Crowe classification (OR = 1.624, 95% CI: 1.165–2.265, *P* = 0.004), osteoporosis (OR = 19.708, 95% CI: 6.089–63.788, *P* < 0.001), greater soft tissue thickness (OR = 1.140, 95% CI: 1.055–1.232, *P* < 0.001), larger NSA (OR = 1.139, 95% CI: 1.070–1.212, *P* < 0.001), and increased femoral offset (OR = 1.095, 95% CI: 1.018–1.177, *P* = 0.010) were significantly associated with historical DAA selection. Female sex was associated with a lower likelihood of DAA selection (OR = 0.395, 95% CI: 0.170–0.914, *P* = 0.030). No significant associations were observed for BMI, diagnosis, rheumatoid arthritis, prior surgery, LCEA, or Dorr classification (all *P* > 0.05). These findings describe historical surgeon preferences within the study cohort and should not be interpreted as evidence supporting the superiority of either surgical approach.

**Table 2:** Univariate logistic regression analysis of preoperative factors associated with historical selection of DAA versus PLA.

Variable	β	OR	95%CI	*P* value
Age	0.124	1.133	1.070–1.199	<0.001
Sex	−0.929	0.395	0.170–0.914	0.030
BMI	−0.023	0.977	0.863–1.107	0.719
Diagnosis	0.302	1.353	0.840–2.180	0.214
Crowe classification	0.485	1.624	1.165–2.265	0.004
Osteoporosis	2.981	19.708	6.089–63.788	<0.001
Rheumatoid arthritis	1.106	3.021	0.677–13.471	0.147
Prior surgery	0.187	1.205	0.353–4.116	0.766
LCEA	0.019	1.019	0.882–1.177	0.799
Soft tissue thickness	0.131	1.140	1.055–1.232	<0.001
Dorr classification	0.192	1.211	0.587–2.498	0.604
NSA	0.130	1.139	1.070–1.212	<0.001
Femoral offset	0.090	1.095	1.018–1.177	0.01

### 3.3 Comparison of different machine learning algorithms

To further validate the performance of the XGBoost model, this study constructed and compared three machine learning models: traditional Logistic Regression (LR), Random Forest (RF), and XGBoost. The results showed that the Random Forest model achieved the highest AUC value of 0.958 in the test set, with an accuracy of 0.90. The XGBoost model attained an AUC of 0.917 and an accuracy of 0.80, while the Logistic Regression model exhibited relatively inferior performance, with an AUC of 0.865. The detailed results are presented in Table 3. Although the Random Forest model marginally outperformed XGBoost in certain metrics, the XGBoost model demonstrated superior stability and clinical interpretability in cross-validation, calibration analysis, and SHAP-based interpretability analysis. Therefore, XGBoost was ultimately selected as the primary predictive model in this study. The detailed results are presented in Table 4.

**Table 3:** Comparison of different ML models.

Model	AUC	Accuracy	Precision	Recall	F1-score
Logistic Regression	0.865	0.75	0.667	0.75	0.706
Random Forest	0.958	0.90	1.00	0.75	0.857
XGBoost	0.917	0.80	0.75	0.75	0.75

**Table 4:** Five-fold cross-validation performance of machine learning models.

Model	Mean CV AUC	SD
Logistic Regression	0.920	0.036
Random Forest	0.956	0.030
XGBoost	0.949	0.028

### 3.4 Machine learning model construction and performance evaluation

**Figure 2:**
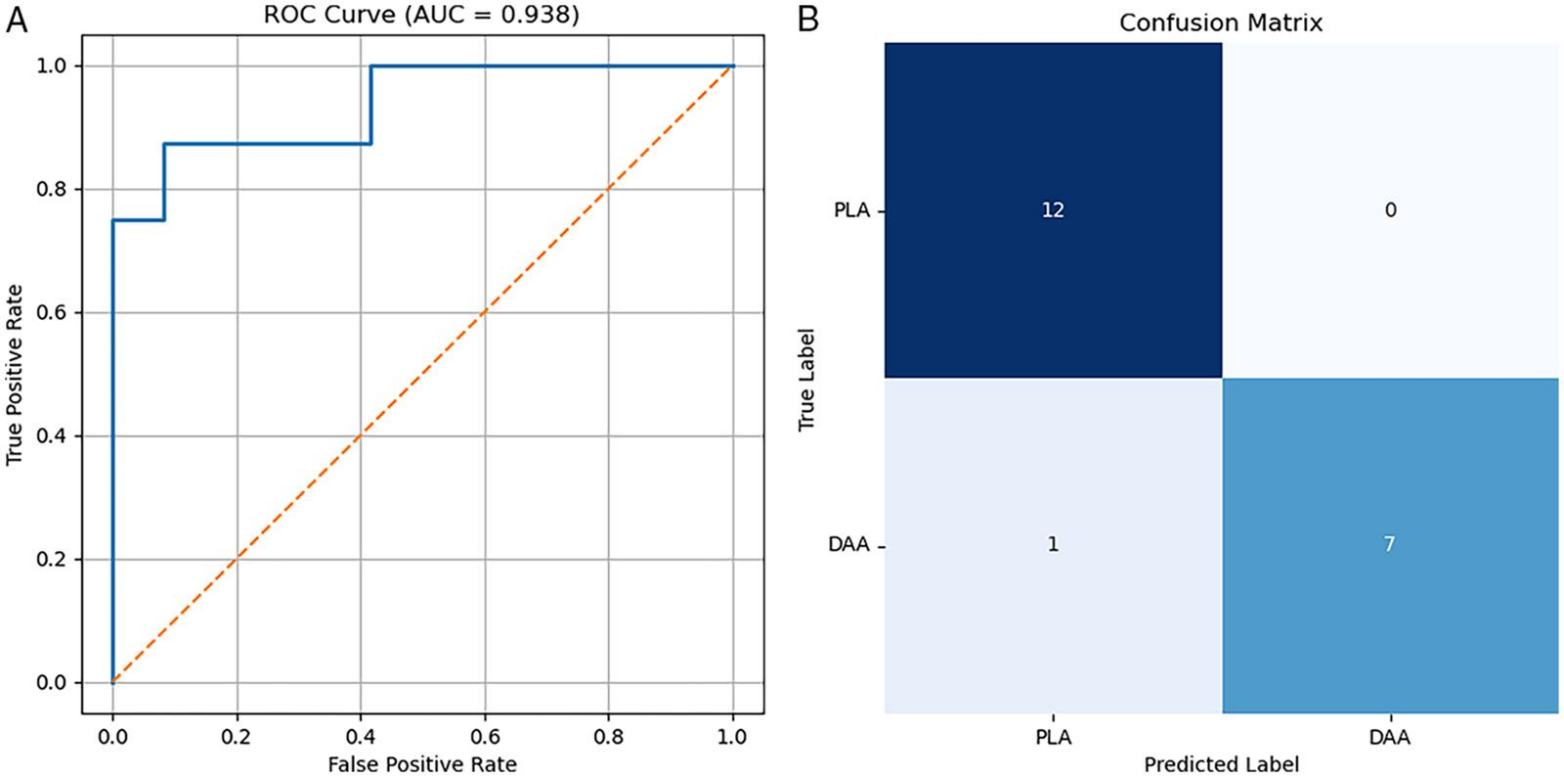
**(A,B)** ROC curve and model confusion matrix diagram.

An XGBoost algorithm was employed to construct a predictive model for determining the surgical approach in THA. The dataset was randomly divided into a development set and a test set at an 8:2 ratio, resulting in 78 cases in the development set and 20 cases in the test set. To address the class imbalance between the two groups, the SMOTE was applied to the development set during the training phase. Hyperparameter tuning was performed using GridSearchCV with five-fold cross-validation and AUC as the optimization metric. The search space included n_estimators (50–200), max_depth (2–5), learning_rate (0.01–0.2), subsample (0.8–1.0), and colsample_bytree (0.8–1.0).The optimized model achieved an AUC of 0.938 and an accuracy of 0.900 on the test set, indicating favorable predictive performance, as illustrated in Figure 2A. The overall accuracy was 90.00%. The confusion matrix results demonstrated that, among the 20 test samples, the model correctly identified 12 patients who underwent the PLA, with only one misclassification, and correctly identified 7 patients who underwent the DAA, with one misclassification, as shown in Figure 2B. The repeated cross-validation analysis further demonstrated that model performance remained generally favorable, with a mean AUC of 0.923 across 100 validation iterations. However, the empirical 95% confidence interval ranged from 0.761 to 1.000, indicating that performance estimates were influenced by data partitioning. This variability is not unexpected given the relatively small sample size and highlights the preliminary nature of the current findings. Therefore, larger multicenter cohorts and external validation studies are required before broader generalizability can be established.

### 3.5 Feature importance and SHAP interpretability analysis

**Figure 3:**
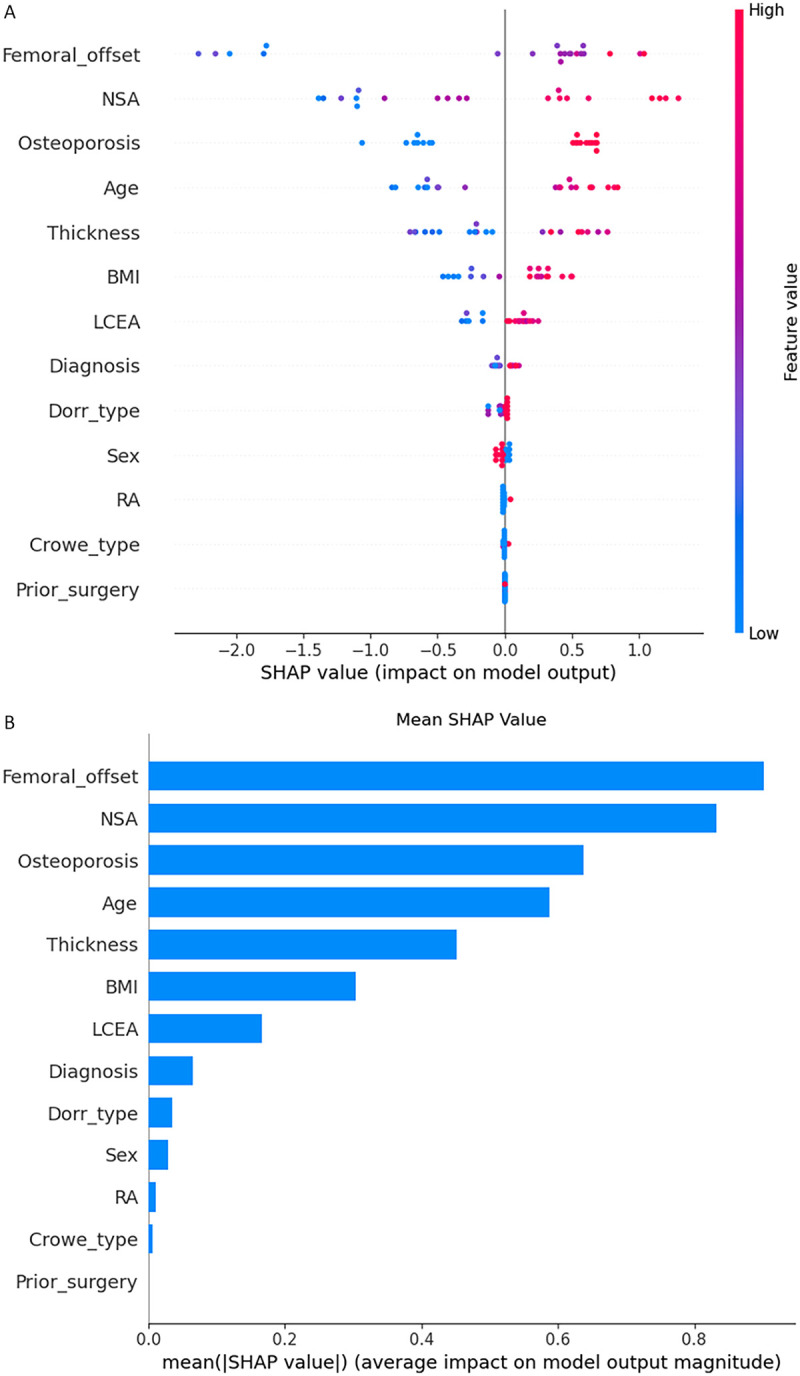
**(A,B)** Model shap chart.

To enhance the interpretability of the model, this study further employed the SHAP method to analyze the contribution of each variable to the model's predictions. The SHAP summary plot (Figures 3A,B) demonstrated that femoral offset, femoral neck-shaft angle (NSA), osteoporosis, age, and soft tissue thickness were the primary variables influencing the model's predictions. Specifically, a larger femoral offset, a larger NSA, advanced age, and thicker soft tissue tended to drive the model toward predicting the DAA. Conversely, a smaller femoral offset and a smaller NSA were more inclined to predict the PLA. These findings suggest that proximal femoral anatomical characteristics and bone quality may be critical factors influencing surgeon preference toward a specific surgical approach in THA.

### 3.6 Results of 5-fold cross-validation

**Figure 4:**
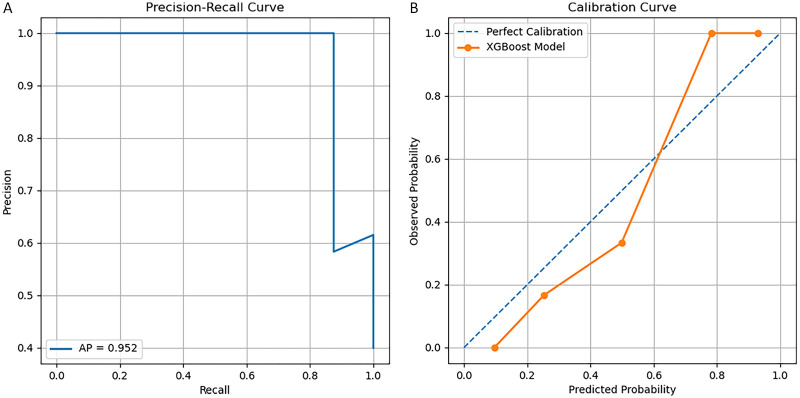
**(A,B)** Model PR curve and model calibration capability curve.

To further evaluate the stability and generalization capability of the model, a 5-fold cross-validation was employed for internal validation in this study. The AUC values obtained from the five folds were 0.747, 0.802, 0.958, 1.000, and 0.976, respectively, with a mean AUC of 0.897 ± 0.102. The corresponding accuracies for each fold were 0.850, 0.750, 0.850, 0.895, and 0.947, yielding an average accuracy of 0.858 ± 0.065. The precision-recall (PR) curves generated from the 5-fold cross-validation are presented in Figure 4A. These results indicate that the model consistently maintained high predictive performance under different data partitioning conditions, demonstrating favorable stability and generalization capability. To further quantify model uncertainty, repeated stratified 5-fold cross-validation with 20 repetitions (100 validation iterations) was performed. The model achieved a mean AUC of 0.923 with a standard deviation of 0.061. The empirical 95% confidence interval ranged from 0.761 to 1.000. Although the average discriminative performance remained favorable, the relatively wide confidence interval indicates a degree of sensitivity to data partitioning, which is likely attributable to the limited sample size of the present study.

### 3.7 Analysis of model calibration ability

To evaluate the consistency between the predicted probabilities of the model and the actual clinical outcomes, a calibration curve analysis and Brier score evaluation were conducted in this study. The calibration curve demonstrated that the model's predicted probability curve was generally close to the ideal 45° reference line, indicating a high level of agreement between the predicted probabilities and the observed outcomes. Furthermore, the Brier score of the model was 0.0898, suggesting satisfactory overall calibration performance and high reliability of the predicted probabilities, as illustrated in Figure 4B. Collectively, these results indicate that the model not only possesses strong discriminative ability but also exhibits favorable reliability in clinical probability prediction, thereby providing preliminary insights for the selection of the preoperative approach in THA.

## 4 Discussion

The selection of the surgical approach for THA has long been a subject of debate. However, in clinical practice, this decision has never truly arisen from a neutral of options; rather, it is the result of a deep integration of the surgeon's personal experience and the patient's unique anatomical characteristics. From a fundamental perspective, the ideal candidate for the DAA should simultaneously possess bone mineral density, acetabular morphology, and femoral geometry that are suitable for standard technical procedures, while also maintaining a body weight within the normal range (8). For patients who do not meet these criteria, such as those with a synthesis of retained internal fixation hardware, complex hip deformities, or morbid obesity (BMI > 40), the posterior or lateral approaches are generally more appropriate (9–11). It is within this context of multi-approach trade-offs that the core premise of the present study was established: the selection of a surgical approach is not arbitrary but is instead a structured decision driven by a set of quantifiable patient characteristics. Importantly, the present model does not establish which surgical approach is objectively superior for a given patient. Rather, it reflects how experienced surgeons incorporated patient anatomical and clinical characteristics into real-world approach selection. The modeling results of this study support this judgment. As presented in Table 5, the majority of existing studies are conventional clinical comparative studies or meta-analyses, lacking predictive models based on machine learning and interpretability analyses. In contrast, the present study further integrated clinical and imaging variables to establish an interpretable predictive model for THA approach selection, thereby offering a novel perspective for individualized surgical decision-making.

**Table 5:** Previous studies have mainly focused on the comparison of the perioperative efficacy and complications between DAA and PLA.

Study	Year	Study design	Sample size	Clinical Scenario	AI/ML Model	Validation	Explainability analysis	Calibration analysis	Main Findings
Chen et al. (12)	2022	Systematic review and meta-analysis	34,873 THAs	DAA vs PLA clinical outcomes	No	No	No	No	DAA showed faster early recovery but longer operative time
Zhao et al. (13)	2017	Randomized controlled trial	101 THAs	Early functional recovery	No	No	No	No	DAA improved early Postoperative recovery
Wang et al. (14)	2024	Meta-analysis	>10,000 THAs	DAA vs PLA efficacy	No	No	No	No	DAA reduced postoperative pain and hospital sta
Zheng et al. (7)	2025	Retrospective cohort	3D CT-based THA planning	AI-assisted implant positioning	Partial AI assistance	No	No	No	AI-assisted planning improved implant positioning accuracy
Present study	2026	Dual-center retrospective ML study	98 THAs	Individualized THA approach prediction	XGBoost	Five-fold CV	SHAP	Yes	Developed an interpretable ML model for individualized DAA/PLA selection with AUC 0.938

Among the variables incorporated into the predictive model of this study, patient age demonstrated a significant predictive weight for surgical approach selection, with older patients exhibiting a higher likelihood of undergoing the DAA. This pattern is highly consistent with current evidence-based findings regarding the application of DAA in the elderly population. A 2022 meta-analysis encompassing 1,284 elderly patients who underwent THA revealed that, compared with the PLA, DAA conferred faster functional recovery and an earlier return to daily activities (15). From a mechanistic perspective, the appeal of DAA for older patients partly stems from the preservation of the posterior joint capsule, thereby reducing the risk of dislocation in a population with already diminished muscle protective capacity. Furthermore, DAA does not necessitate strict postoperative restrictions on hip range of motion, facilitating early weight-bearing ambulation—a clinically profound benefit for very elderly patients with concurrent femoral neck fragility fractures or multisystem comorbidities (16). Of note, age does not function as an independent determinant: within the present cohort, age consistently exhibited a synergistic effect with proximal femoral bone morphology parameters, rather than exerting an isolated influence. This observation is biologically plausible, as both bone quality and medullary canal geometry undergo predictable degenerative changes with advancing age. Such interaction effects among variables warrant careful consideration when applying this model across cohorts of different age groups.

The Dorr classification of proximal femoral bone morphology emerged as one of the most influential predictors of surgical approach within the study dataset, a finding that carries direct clinical logic. Dorr type A femurs are characterized by thick cortical walls and a narrow, funnel-shaped medullary canal, offering excellent bone stock for cementless implant fixation. Their proximal “funnel” morphology is also highly compatible with the confined operative space encountered during femoral-side exposure via the DAA (17). In contrast, Dorr type C femurs—exhibiting a wide, straight medullary canal (“stovepipe” morphology) with markedly thin cortices—present a distinctly different surgical scenario. Dense bone (Dorr types A–B) has traditionally been regarded as the ideal substrate for cementless fixation, whereas osteoporotic bone (Dorr types B–C) is more suitable for optimized cemented fixation techniques (18). The clinical risk associated with Dorr type C bone should not be underestimated: a multicenter study involving 709 THA cases demonstrated a graded increase in periprosthetic femoral fracture rates with cementless implants according to Dorr classification—2.3% for type A, 3.7% for type B, and as high as 15.9% for type C (*P* < 0.001). Logistic regression analysis further confirmed Dorr type C bone as an independent risk factor for this complication (19). Faced with such high-risk bone quality, surgeons tend to favor the posterolateral approach when managing Dorr type C cases, as it provides more extensive exposure of the proximal femur, thereby enabling safe reaming and cement placement within the challenging medullary canal (20).The concordance between these literature findings and the predictions derived from the current model indirectly validates the biological plausibility of the predictive logic.

The femoral NSA and femoral offset collectively determine the three-dimensional geometric configuration of the proximal femur, thereby directly influencing the accessibility of the femoral medullary canal through different surgical approaches. For the DAA, a larger valgus NSA confers an operational advantage: it positions the femoral medullary canal relatively more anteriorly, thereby reducing the soft tissue tension that must be overcome during external rotation and extension maneuvers at the stage of femoral preparation (21). Conversely, a decreased NSA combined with a reduced femoral offset results in a more deeply located medullary canal within the thigh, significantly narrowing the operational window for DAA and increasing the mechanical difficulty of reaming. This anatomical feature has been established as a relative contraindication for DAA (22). The aforementioned geometric parameters not only influence the ease of prosthesis implantation but are also closely associated with the risk of nerve injury. A dedicated study investigating risk factors for lateral femoral cutaneous nerve (LFCN) injury in DAA–THA identified a smaller femoral offset as a significant independent predictor in multivariate analysis, suggesting that insufficient offset not only increases the technical difficulty of implant placement but also elevates the risk of nerve injury due to exacerbated soft tissue retraction during exposure (23). The implications of these findings for the modeling approach in the present study are as follows: the NSA and femoral offset are not ancillary radiological parameters but rather quantitative surrogate markers of operative difficulty, capable of exerting a substantive impact on the probability distribution of approach selection. Recent studies have further highlighted the importance of pelvic morphology in determining the technical feasibility of the direct anterior approach. In particular, the spatial relationship between the anterior superior iliac spine (ASIS) and the greater trochanter has emerged as an important surrogate marker of surgical exposure. A large retrospective study involving 960 DAA procedures demonstrated that a shorter ASIS-to-greater trochanter distance (DAA plane) was significantly associated with prolonged operative time, suggesting a narrower working corridor for femoral preparation (24). Similarly, reduced ASIS–greater trochanter spatial parameters have been linked to increased blood loss and a higher rate of surgical complications. These findings support the concept that pelvic morphology influences the accessibility of the proximal femur during DAA surgery. From a biomechanical perspective, a prominent ASIS or a deep V-shaped pelvic configuration may mechanically restrict anterior femoral elevation and external rotation during femoral exposure (25). Consequently, even when patient age and bone quality are favorable, limited operative space created by pelvic morphology may reduce the practicality of DAA and shift surgeon preference toward a posterolateral approach (26). These anatomical parameters may partially explain surgeon-specific approach selection behavior and deserve further investigation in future predictive models.

One of the long-standing core criticisms of machine learning models in clinical medicine pertains to the opacity of their predictive processes and the inability of end users to trace the rationale behind a specific prediction made by the model. To address this issue, the present study employed the SHAP method, which is grounded in game theory (27). In orthopedic surgery, such granular interpretability carries direct clinical value. A large-scale evaluation of interpretability methods involving 517,826 primary total joint arthroplasties demonstrated a directional consistency of up to 97.8% between SHAP attribution results and partial dependence plots (PDPs), thereby methodologically validating the reliability of SHAP as an explanatory tool for orthopedic predictive models (28). In the SHAP summary plot of the present model, Dorr classification, age, NSA, and femoral offset ranked among the top features in terms of importance. This hierarchical structure aligns closely with established surgical clinical reasoning, rather than contradicting it. Such consistency is not coincidental: it indicates that the decision boundaries learned by the model are clinically plausible, rather than reflecting spurious correlations in the training data. For clinical orthopedic surgeons, the SHAP-driven model output can serve as a structured reference checklist, visually presenting the anatomical basis underlying each approach recommendation, thereby facilitating shared decision-making between clinicians and patients (29).It should be emphasized that SHAP values quantify the contribution of variables to model predictions rather than causal effects on surgeon decision-making. Variables identified as important by SHAP may reflect patterns present within the training data and should not be interpreted as independent determinants of surgical approach selection. Further prospective studies are required to investigate causal relationships.

In the evaluation of a model's clinical applicability, discriminative ability alone is insufficient as a sole criterion. Calibration analysis is specifically designed to address this issue and is now widely recognized as an indispensable complement to the AUC in the assessment of medical prediction tools (30). In the present study, the calibration curve demonstrated good agreement between predicted and observed probabilities across the entire risk spectrum, indicating that the model's output can be interpreted directly as clinical probabilities rather than merely as ordinal ranking scores (31). Notably, a model may exhibit strong AUC performance while simultaneously suffering from substantial calibration bias—a discrepancy that remains entirely undetectable through pure discrimination metrics. In this study, the Brier score remained low across both internal cross-validation folds and the independent test set, suggesting that the model does not display systematic overconfidence or excessive conservatism in its predictions during generalization. However, it should be noted that the absolute value of the Brier score is influenced by the prevalence of the outcome event in the sample; thus, direct comparisons across different cohorts should be interpreted with caution (32). Before broader generalizability can be inferred, external validation using multicenter datasets with diverse case compositions is still required. In essence, the machine learning model learns this implicit clinical decision-making pattern. Furthermore, the model was validated using five-fold cross-validation and calibration analysis, and the results demonstrated satisfactory stability and calibration ability. Both Random Forest and XGBoost demonstrated excellent predictive performance in cross-validation and independent testing. Despite promising discrimination performance, the results should be interpreted cautiously because of the relatively small sample size and limited test cohort. The repeated cross-validation analysis further demonstrated that model performance remained generally favorable, with a mean AUC of 0.923 across 100 validation iterations. However, the empirical 95% confidence interval ranged from 0.761 to 1.000, indicating that performance estimates were influenced by data partitioning. This variability is not unexpected given the relatively small sample size and highlights the preliminary nature of the current findings. Therefore, larger multicenter cohorts and external validation studies are required before broader generalizability can be established.

This study has several limitations. First, due to its retrospective nature, there is an inherent risk of selection bias, and the sample size was relatively limited, particularly the small number of cases in the DAA group, which may affect the generalizability of the model. Second, although data were collected from two hospitals and involved two senior arthroplasty surgeons, the model lacks broader external validation; its applicability across different regions and surgeons requires further evaluation. Additionally, some of the imaging parameters included in this study were primarily derived from two-dimensional x-ray measurements, which cannot fully capture the complex three-dimensional anatomy of the hip joint. Finally, the model was designed to assist in preoperative surgical approach decision-making and is not intended to replace the comprehensive judgment of clinicians. Future studies with larger sample sizes, multicenter and prospective designs, incorporating three-dimensional imaging and additional perioperative indicators, are warranted to further optimize the model's performance. Most importantly, the outcome label used in this study represented the historical surgical approach selected by surgeons rather than objectively validated optimal treatment strategies. Therefore, the present model should not be interpreted as a tool for recommending the best surgical approach for individual patients. Instead, it should be regarded as an exploratory analysis of real-world surgical selection behavior. In addition, no postoperative functional outcomes, complications, revision rates, or patient-reported outcome measures were included in the current analysis.

## 5 Conclusion

Historical surgeon selection of DAA versus PLA in THA was associated with several patient-specific clinical and radiographic characteristics, including age, proximal femoral morphology, and bone quality. The present machine learning model was able to characterize real-world surgical selection patterns with reasonable discriminative performance. However, the model should not be interpreted as identifying the objectively optimal surgical approach for individual patients.

## Data Availability

The original contributions presented in the study are included in the article/Supplementary Material, further inquiries can be directed to the corresponding author.
